# Physical Function Trajectory among High-Functioning Long-Term Care Facility Residents: Utilizing Japanese National Data

**DOI:** 10.3390/geriatrics9050123

**Published:** 2024-09-19

**Authors:** Kasumi Ikuta, Maiko Noguchi-Watanabe, Miya Aishima, Tatsuhiko Anzai, Kunihiko Takahashi, Sakiko Fukui

**Affiliations:** 1 Department of Home Health and Palliative Care Nursing, Tokyo Medical and Dental University, 1-5-45 Yushima, Bunkyo-Ku, Tokyo 113-8519, Japan; 2 Department of Biostatistics, M&D Data Science Center, Tokyo Medical and Dental University, 2-3-10 Kandasurugadai, Chiyoda-Ku, Tokyo 101-0062, Japan

**Keywords:** long-term care information system, physical function trajectory, high-functioning long-term care facility residents, multicenter retrospective cohort study

## Abstract

Physical function trajectory (PFT) is associated with mortality and hospitalization risks. We aimed to identify and compare the PFTs of newly admitted high-functioning older adults during their first six months at long-term care (LTC) facilities. In this multicenter retrospective cohort study, we included newly admitted high-functioning older adults (Barthel index > 60) from 47 Japanese LTC facilities. The primary outcome was physical function changes after admission. Data were collected from the Long-Term Care Information System for Evidence (LIFE), which monitored LTC facility residents’ function between 1 July 2021 and 28 February 2023. A group-based trajectory model and binomial logistic regression analyses were applied to identify and compare residents’ PFTs. Among the 718 residents included, the average age was 85.69 years and 64.5% were female. PFTs were classified as maintenance (66.0%), improvement (9.5%), slight decline (16.6%), and large decline (7.9%). The improvement group had significantly fewer residents who expressed a lack of interest in daily activities (odds ratio (OR) 0.45; 95% confidence interval (CI) 0.21–0.97) compared to the maintenance group. The large decline group had significantly more residents with a low BMI at admission (OR 2.42; 95% CI 1.29–4.55) and residents who did not use dentures (OR 0.49; 95% CI 0.26–0.95), compared to the maintenance group. Considering future PFTs may aid the development of care plans and the provision of appropriate interventions. Moreover, utilizing existing data has the potential to maintain residents’ physical independence and enhance the quality of care without burdening residents themselves or staff.

## 1. Introduction

Population aging is progressing worldwide, including in Japan [1,2], where the percentage of older adults is rapidly increasing. By 2050, it is estimated that the global older adult population will have doubled, significantly impacting healthcare and long-term care (LTC) systems [3]. Older adults experience significant declines in physical functions [3,4], with approximately 64% having at least one functional limitation, the prevalence of which increases with age [5]. These issues are rising globally as the population ages, necessitating enhanced LTC strategies [6]. Declining physical function is associated with the risk of death, hospital admission, and impaired quality of life [7,8], requiring careful observation and evaluation. Physical function trajectory (PFT), which reflects the rate of change in physical function, is used to identify individuals requiring intervention as a priority [9]. At LTC facilities, staff comprehensively assess each resident at admission and devise individualized care plans based on the anticipated changes in health status. This initial stage is crucial and can significantly impact health outcomes [9]. Therefore, staff equipped with PFT information can develop appropriate interventions and end-of-life care plans.

Despite its importance, studies on PFT among LTC facility residents have shown many inconsistencies [10,11,12]. These inconsistencies often arise owing to variations in participant backgrounds [10,11]. To date, only one study has used PFTs for six months from the time of admission, categorizing a single decline group [12]. Another study [13] suggested that factors associated with physical function decline differ according to initial physical function levels at admission, indicating the potential existence of multiple decline groups in PFTs.

We focused on newly admitted residents with high physical function. They are proven to have the potential to improve or maintain physical function through appropriate interventions [14]. Previous studies have examined groups, such as independent community residents [15] and LTC facility residents who can move without any walking aid [10]; however, studies on newly admitted LTC facility residents with high physical function are lacking. Thus, we considered it useful to identify PFTs in newly admitted residents with high physical function who are likely to show the effects of interventions. 

Additionally, we used the Long-Term Care Information System for Evidence (LIFE), launched by the Japanese government in 2021 [14,15], to evaluate the physical function of LTC facility residents. Japan has one of the most rapidly aging populations globally [16], with approximately 30% of its 125 million people aged >65 years in 2022 [17]. Increasing the number of care workers is challenging, as the productive age population is decreasing [18]. Information on effective long-term evidence-based care in Japan could provide useful insights for optimizing LTC provision in other countries with limited human and financial resources; however, current research using LIFE data is limited [13,19,20]. Therefore, we aimed to identify and compare, using LIFE data, the PFTs of newly admitted high-functioning LTC facility residents during the first six months of admission.

## 2. Materials and Methods

### 2.1. Study Design, Setting, and Population

In this multicenter retrospective cohort study, we utilized routinely collected LIFE data from high-functioning LTC facility residents (Barthel index (BI) > 60 [21]) across 47 urban LTC facilities in Japan between 1 July 2021 and 28 February 2023. The targeted LTC facilities were classified as specified facilities in LTC insurance services [22]. These facilities provide daily living assistance and basic medical care through staff, including care workers and nurses (one nurse for every 30–50 people) [23], but they are not medical facilities, nor do they have physicians. The observational period spanned six months. We excluded residents with missing LIFE data or whose duration of stay was shorter than six months.

This study was approved by the Medical Research Ethics Committee of Tokyo Medical and Dental University (approval no. M2021-285). We adopted an opt-out system because this was a retrospective study. We displayed documents detailing the study’s purpose, methodology, privacy policy, and contact information for the researchers on each LTC facility’s website, and then mailed these details to the residents’ family members. 

### 2.2. Data Collection

We obtained monthly LIFE data from LTC facility records. LIFE stores real-world data collected from LTC services, which can be used to promote evidence-based care practice, similar to the Minimum Data Set 3.0. It evaluates disability outcomes and health status, such as physical and cognitive function and nutritional and oral health using international indicators. Approximately 60,000 LTC services, such as special LTC facilities and specified facilities, registered and submitted these data in 2021; this number is expected to increase continually [14]. 

We collected LIFE data for each resident from admission to six months post-admission. The data were entered monthly into the LIFE database by staff involved in resident care and evaluation, such as nurses and care managers. Facility managers instructed staff on input criteria to standardize the LIFE data. Facility managers collected these data from 47 LTC facilities and submitted them to our research team.

### 2.3. Outcome

The study outcome was changes in physical function after admission, measured using the BI [24]. We calculated the difference between BI at admission and monthly for six months after admission (a total of five points) as the change in physical function. The BI is an ordinal scale that measures all aspects of activities of daily living and is used worldwide [25]. Each item was scored, and the total score was determined by summing all items. The BI comprises 10 items: toilet use, chair/bed transfer, personal hygiene, dressing, ambulation, feeding, bowel control, bladder control, self-bathing, and stair climbing (Table S1) [24]. The BI is scored from 0 to 100, with 100 representing complete self-sufficiency and 0 representing complete dependence.

The value of change in physical function at the time of admission was 0 for all participants. A negative change value indicated a decline in physical function from admission, and a positive value indicated improvement.

### 2.4. Exposure

We used the following items for exposure: age, sex, low body mass index (BMI), low cognitive function, eating preferences (soft food), use of dentures, easy choking, items on the BI, the Dementia Behavior Disturbance Scale (DBD), and the Vitality Index (VI). Data were collected from the LTC facility records. Low BMI was defined as <18.5 kg/m^2^.

Low cognitive function was evaluated using the dementia rating employed in the Japanese LTC system [26] and in previous studies [13,27]. Responses were rated on a five-point scale: almost independent, independent if someone looks after them, mild cognitive impairment, moderate cognitive impairment, and severe cognitive impairment. Low cognitive function was defined as mild cognitive impairment and above.

The DBD evaluates the behavioral disturbances common to patients with dementia and comprises 28 original items [28,29]. We used the five-item version to evaluate a lack of interest in daily activities, awakening during the night, accusations, walking around, and repeating the same action [30]. Responses were rated on a five-point scale (never, seldom, occasional, often, and always). Symptom presence was defined as occasional and above.

The VI is a five-item scale assessing vitality related to activities of daily living [31]. LIFE evaluates communication, one of the aspects of the VI, on three levels: vocalizing reciprocal exchanges at will, responsiveness to verbal stimulation, and no cognitive response. Low motivation was defined as a response to verbal stimulation and a lack of cognitive response. 

### 2.5. Statistical Analysis

We created a dataset using the monthly BI for each resident from admission to six months later and calculated the difference between each month’s BI and BI at admission as the change in physical function. To identify PFTs from admission to six months later, we used group-based trajectory modeling—a statistical method for approximating the function of a continuous population distribution of a trajectory of unknown shapes [12,32]. The group-based trajectory model was fitted into two to six groups in a tertiary shape using six points from admission to six months later [10,12,33]. We compared the fit statistics and graphical depictions of the models. First, we checked the changes in Akaike’s information criterion (AIC) and the Bayesian information criterion (BIC) as fit statistics. Subsequently, we checked whether the average posterior probability (AvePP) of all groups was >0.7. Additionally, we confirmed that the odds of correct classification (OCC) were >5 for all groups. The trajectory was considered using these fitness and clinical interpretations, and groups were determined. Finally, a qualitative label was assigned to each trajectory.

Additionally, we assigned residents to the trajectory with the highest posterior probability of belonging. We then described the characteristics of residents for each PFT. Following this, we conducted a binary logistic regression analysis to independently compare the characteristics with the other groups using the maintenance group as a reference. This analysis is useful for determining which trajectory residents are likely to follow based on their information at the time of admission. We used single items of the BI as covariate factors after confirming collinearity. The results were assessed using the Guidelines for Reporting on Latent Trajectory Studies Checklist [32,34]. Data were managed using Python, version 3.8.1 for Mac (Python Software Foundation, Wilmington, DE, USA). All analyses were performed using R version 3.6.3 for Mac (R Foundation for Statistical Computing, Vienna, Austria). Statistical significance was set at *p* < 0.05.

## 3. Results

### 3.1. Participant Selection and Follow-Up

**Figure 1:**
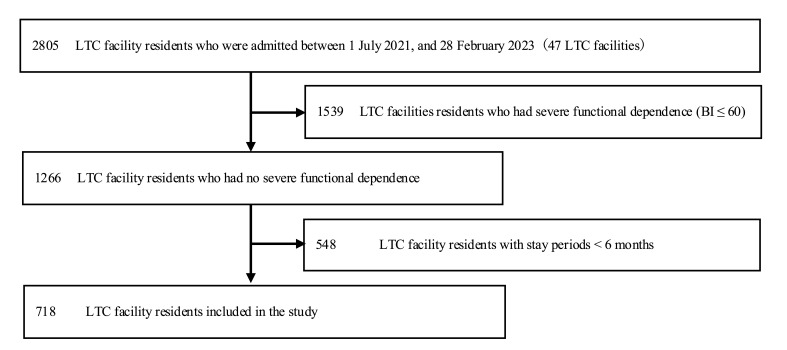
Flow diagram for the inclusion and exclusion of study participants. Notes: LTC, long-term care.

Of the LTC facility residents admitted between 1 July 2021 and 28 February 2023 (n = 2805, 47 LTC facilities), we excluded residents with a BI ≤ 60 (n = 1539), those with stay periods shorter than six months (n = 548), and those with missing LIFE data (n = 0); thus, we included 718 residents in the analysis (Figure 1). Residents who completed the follow-up and those who did not numbered 718 (56.7%) and 548 (43.3%), respectively. Residents who completed the follow-up were older and exhibited lower cognitive and physical function upon admission compared to those who did not complete the follow-up (Table S2). 

### 3.2. Participant Characteristics

Participant characteristics at admission are presented in Table 1. At admission, the average age was 85.69 years (standard deviation = 7.01), 64.5% of participants were female, and 68.5% had low cognitive function. Based on BI scores, 80% required assistance with self-bathing (80.5%) and stair climbing (80.8%). 

### 3.3. Trajectory

We depicted the trajectories of two to six groups (Figure S1). We evaluated the AIC, BIC, proportion of each group, AvePP, and OCC for each trajectory group (Table S3). We chose four groups because we depicted the real trajectory for each participant and considered this result to be clinically interpretable and meaningful (Figures S2 and S3). With three groups, the maintenance group included an improved PFT. With five groups, three decline groups were identified, with only a five-point decline (one-item change on the BI) for the group with the least change. We did not adopt the five-group model as we did not identify a clinically important difference in the five-point decline.

**Figure 2:**
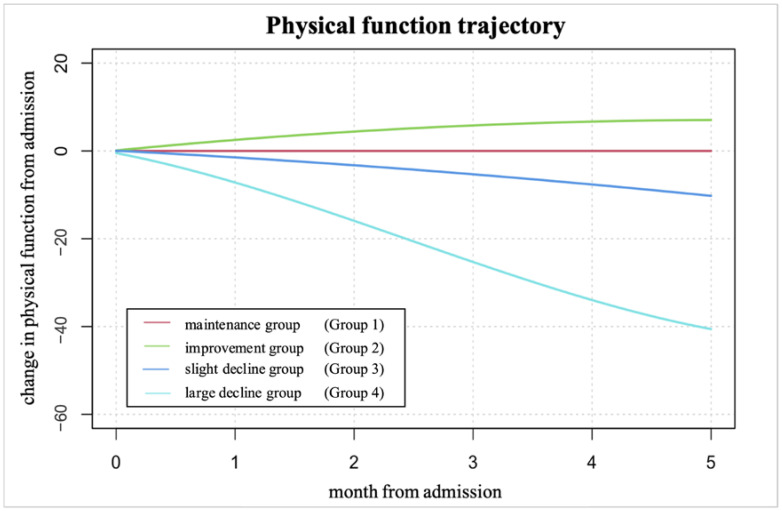
PFTs from admission to six months later. We used group-based trajectory modeling to identify PFTs from admission to six months later. Notes: PFTs, physical function trajectories.

We identified four groups of trajectories (Figure 2): maintenance, improvement, slight decline, and large decline. The AIC and BIC were −660129.2 and −660027.6, respectively. The AvePPs of the four groups ranged from 0.96 to 0.99. The proportions of patients in the maintenance, improvement, slight decline, and large decline groups were 66.0%, 9.5%, 16.6%, and 7.9%, respectively.

### 3.4. Characteristics of Residents for Each Identified PFT

The characteristics of each trajectory are illustrated in Table 2. BI at admission (*p* < 0.001), low BMI (*p* = 0.007), and waking at night (*p* = 0.005) had significant differences between the identified trajectories.

Table 3 shows the characteristics of each PFT group compared to the maintenance group. The improvement group had significantly fewer residents who expressed a lack of interest in daily activities (odds ratio (OR) = 0.45; 95% confidence interval (CI) = 0.21–0.97) compared to the maintenance group. Second, the slight decline group had significantly more residents awake at midnight (OR = 2.21; 95% CI = 1.19–4.10) with low motivation at admission (OR = 1.58; 95% CI = 1.01–2.47) compared to the maintenance group. Finally, the large decline group had significantly more residents with a low BMI at admission (OR = 2.42; 95% CI = 1.29–4.55) and those who did not use dentures (OR = 0.49; 95% CI = 0.26–0.95) compared to the maintenance group.

## 4. Discussion

We examined PFTs among newly admitted LTC facility residents using national LIFE data. This is the first attempt to longitudinally evaluate changes in physical function since 2021, when LIFE was launched in Japan. We identified four trajectories: maintenance, improvement, slight decline, and large decline. Moreover, we described the characteristics at admission using only routinely collected data, allowing us to assess which of the four groups residents were likely to belong to without burdening them and their staff. 

Similar to past studies in which the participants were older adults with independent physical function [10,33], we focused on high-functioning residents and did not include residents with severe or total dependency; therefore, we considered observing multiple decline trajectories. In addition, the proportion of the two decline groups was 24.5%, and that of the improvement group was 9.5%. The decline and improvement groups had a higher proportion than those in a previous study (7.6%) [12] because we detected small changes in physical function and classified participants accordingly. Additionally, we targeted LTC facility residents with high physical functioning; therefore, the condition of many residents may have improved. We considered the potential background of residents with improved physical function—residents were admitted immediately after a fracture or other injury and were in the recovery phase; thus, their physical function may have improved gradually [35]. However, it is important to note that we did not include this information in our study; thus, we were unable to establish these associations definitively. Further research is needed to explore these potential factors in more depth.

Additionally, the residents in the improvement group were less likely to lack interest in daily activities than the maintenance group. Interest in daily activities is associated with positivity and opportunities for activities; therefore, residents’ physical activity may have increased [36]. Previous studies have demonstrated that increasing physical activity through exercise interventions improves and maintains physical function [37]. We assumed that interest and positivity may increase physical activity, leading to improvements in physical function.

The slight decline group was characterized by more frequent awakening at night and lower motivation than the maintenance group. Older adults experience disrupted sleep and increased nocturnal awakening owing to sleep fragmentation and circadian rhythm-related changes which occur with age [38]. LTC facility residents are generally less active than community residents [39], and insufficient daytime activity may prevent adequate night sleep [40]. As this insufficient sleep then leads to decreased daytime activity, we viewed it as indicating that residents’ physical function had declined [41]. Low motivation is a symptom of apathy [42], which may reduce daytime activity. Promoting daytime physical activity could help such residents maintain their physical function by adjusting their life rhythms. 

Residents assigned to the large decline group were more likely to have a low BMI at admission and to not use dentures properly. Many studies have reported an association between low BMI and physical function decline, such as muscle weakness and sarcopenia [4,13,43]. Additionally, improper denture use may decrease food intake and worsen nutritional status [44]. Approximately 55% of LTC facility residents in a previous study and 59.7% in our study could not use dentures properly [45]. Residents in the large decline group may experience difficulties utilizing their dentures effectively, potentially leading to a decrease in their BMI. Optimizing denture use improves chewing ability [46]; therefore, oral function must be carefully assessed. 

Our findings have practical implications. Comprehending PFTs and their characteristics for those classified in the declining group enables proactive intervention and prediction. Given the recent shortage of caregivers, PFT assessments can help identify residents in need of closer monitoring. Moreover, such assessments can help residents and their families to invest in better social care equipment and to psychologically prepare for physical function decline. However, further research is necessary to determine whether interventions identified in previous studies can alter PFTs.

The strength of our study lies in two key aspects. First, employing existing data, such as LIFE, enables longitudinal assessment of PFTs, unveiling new insights without the need for additional data collection. This approach is replicable across facilities, enabling the longitudinal evaluation of various aspects beyond PFTs and serving as a quantitative indicator assessing facility efforts. Second, we focused on high-functioning LTC facility residents and proposed the existence of multiple decline patterns within a group previously categorized as a single entity [12]. To our knowledge, this is the first study to concentrate on high-functioning LTC facility residents and evaluate PFTs from admission using LIFE.

## 5. Limitations

Despite its strengths, this study has some limitations. First, the generalizability is limited, as the 47 participating LTC facilities are owned by the same corporation and located in urban areas. Residents in urban areas may enter LTC facilities at a younger age than those in rural areas and, therefore, have better physical functioning. A multi-facility study including rural areas could improve generalizability. Second, the results did not sufficiently reflect the level of physical function at admission, as we indicated the PFT using the amount of change; thus, the group of trajectories may have been underestimated. We attempted to minimize this by focusing on residents with high physical function and analyzing data adjusted for single BI items at admission. Third, we excluded participants who died, left, or were admitted to the hospital less than six months after admission; therefore, not all trajectories may have been depicted. We found that LTC facility residents who completed follow-up and those who did not had significantly different ages, BI at admission, and cognitive function. Future studies should include participants who were excluded from this study. Fourth, as LIFE data were collected by multiple LTC facility staff members, this study may contain a measurement bias. However, LIFE specialists consulted the target facilities when necessary to minimize this bias. Future studies should unify the measurements. Fifth, we did not examine medical information reported to be associated with physical function decline [9,11,25]; we instead focused on utilizing LIFE data. As LTC facilities in Japan are not medical facilities, collecting medical data is difficult. Evaluating only routinely collected data may increase feasibility and utilization possibilities at LTC facilities. Despite these limitations, this study has the advantage of evaluating PFTs using nationally standardized LIFE data, which can be used to identify the characteristics at admission and to promote the implementation of optimal LTC.

## 6. Conclusions

We examined the PFTs of residents of 47 Japanese LTC facilities, classifying them into 4 groups: maintenance, improvement, slight decline, and large decline. The large decline group had significantly more residents with low BMI at admission and no denture usage compared to the maintenance group. We used routinely collected LIFE real-world data, allowing for efficient assessment without burdening residents/staff. By tracing PFTs from admission, these results provide information to facilitate appropriate interventions and care for residents.

## Figures and Tables

**Table 1:** Characteristics of LTC facility residents at admission.

N	718
Age (mean (SD))	85.69 (7.01)
Female (%)	463 (64.5)
Low BMI * (%)	179 (24.9)
Low cognitive function ‡ (%)	492 (68.5)
History of aspiration pneumonia (%)	7 (1.0)
Diagnosis of dementia (%)	268 (37.3)
Physical function requiring assistance	
Feeding (%)	19 (2.6)
Chair/bed transfer (%)	77 (10.7)
Personal hygiene (%)	125 (17.4)
Toilet (%)	124 (17.3)
Self-bathing (%)	578 (80.5)
Ambulation (%)	298 (41.5)
Stair climbing (%)	580 (80.8)
Dressing (%)	198 (27.6)
Bowel control (%)	223 (31.1)
Bladder control (%)	126 (17.5)
Eating preference (soft food) (%)	76 (10.6)
Using dentures (%)	289 (40.3)
Choking easily (%)	36 (5.0)
One item of the Vitality Index	
Low motivation (%)	300 (41.8)
Five items of the DBD-13	
Lack of interest in daily activities (%)	196 (27.3)
Waking at night (%)	121 (16.9)
Making an accusation (%)	98 (13.6)
Walking around (%)	159 (22.1)
Repeating the same action (%)	122 (17.0)

Notes: LTC, long-term care, SD, standard deviation; BMI, body mass index; DBD, Dementia Behavior Disturbance. * Low BMI was defined as <18.5 kg/m^2^; ‡ low cognitive function was evaluated using the dementia rating.

**Table 2:** Characteristics of LTC facility residents at admission by identified PFTs.

	Group 1	Group 2	Group 3	Group 4	*p*-Value
	Maintenance Group	Improvement Group	Slight Decline Group	Large Decline Group	
N	474	68	119	57	
Barthel index at admission	82.83 (10.56)	76.76 (9.13)	82.06 (10.56)	82.63 (11.22)	<0.001
Age (mean (SD))	85.48 (7.37)	86.03 (7.06)	85.95 (6.22)	86.49 (5.27)	0.688
Female (%)	305 (64.3)	44 (64.7)	80 (67.2)	34 (59.6)	0.807
Low BMI * (%)	113 (23.8)	11 (16.2)	31 (26.1)	24 (42.1)	0.007
Low cognitive function ‡ (%)	320 (67.5)	46 (67.6)	83 (69.7)	43 (75.4)	0.660
Physical function requiring assistance					
Feeding (%)	7 (1.5)	4 (5.9)	5 (4.2)	3 (5.3)	0.048
Chair/bed transfer (%)	46 (9.7)	13 (19.1)	10 (8.4)	8 (14.0)	0.077
Personal hygiene (%)	76 (16.0)	15 (22.1)	26 (21.8)	8 (14.0)	0.292
Toilet (%)	75 (15.8)	18 (26.5)	22 (18.5)	9 (15.8)	0.177
Self-bathing (%)	380 (80.2)	62 (91.2)	93 (78.2)	43 (75.4)	0.097
Ambulation (%)	190 (40.1)	42 (61.8)	47 (39.5)	19 (33.3)	0.003
Stair climbing (%)	384 (81.0)	61 (89.7)	91 (76.5)	44 (77.2)	0.145
Dressing (%)	120 (25.3)	28 (41.2)	36 (30.3)	14 (24.6)	0.042
Bowel control (%)	137 (28.9)	29 (42.6)	43 (36.1)	14 (24.6)	0.049
Bladder control (%)	76 (16.0)	14 (20.6)	23 (19.3)	13 (22.8)	0.469
History of aspiration pneumonia (%)	4 (0.8)	1 (1.5)	1 (0.8)	1 (1.8)	0.888
Diagnosis of dementia (%)	174 (36.7)	23 (33.8)	43 (36.1)	28 (49.1)	0.273
Eating preference (soft food) (%)	44 (9.3)	9 (13.2)	16 (13.4)	7 (12.3)	0.465
Using dentures (%)	201 (42.4)	26 (38.2)	45 (37.8)	17 (29.8)	0.273
Choking easily (%)	20 (4.2)	3 (4.4)	7 (5.9)	6 (10.5)	0.212
One item of the Vitality Index					
Low motivation (%)	186 (39.2)	28 (41.2)	57 (47.9)	29 (50.9)	0.169
Five items of the DBD-13					
Not showing interest (%)	134 (28.3)	13 (19.1)	30 (25.2)	19 (33.3)	0.281
Awaking at midnight (%)	67 (14.1)	9 (13.2)	29 (24.4)	16 (28.1)	0.005
Making an accusation (%)	59 (12.4)	9 (13.2)	19 (16.0)	11 (19.3)	0.444
Walking around (%)	99 (20.9)	14 (20.6)	29 (24.4)	17 (29.8)	0.420
Repeating an action (%)	77 (16.2)	8 (11.8)	23 (19.3)	14 (24.6)	0.233

Notes: LTC, long-term care; PFTs, physical function trajectories; SD, standard deviation; BMI, body mass index; DBD, Dementia Behavior Disturbance. * Low BMI was defined as <18.5 kg/m^2^; ‡ low cognitive function was evaluated using the dementia rating.

**Table 3:** Characteristics of each PFT group *.

	Improvement Group (G2)		Slight Decline Group (G3)		Large Decline Group (G4)	
	OR (95% CI)	*p*-Value	OR (95% CI)	*p*-Value	OR (95% CI)	*p*-Value
Age (years)	1.01 (0.97–1.05)	0.781	1.01 (0.98–1.04)	0.487	1.04 (0.99–1.09)	0.095
Female	1.13 (0.63–2.02)	0.693	1.19 (0.75–1.89)	0.461	0.60 (0.32–1.14)	0.117
Low BMI ‡	0.54 (0.26–1.12)	0.097	0.94 (0.58–1.53)	0.802	2.42 (1.29–4.55)	0.006
Diagnosis of dementia	0.91 (0.48–1.73)	0.768	0.82 (0.50–1.34)	0.422	1.63 (0.80–3.32)	0.180
Eating preference (soft food)	1.12 (0.43–2.90)	0.815	1.46 (0.72–2.95)	0.293	1.00 (0.34–2.95)	0.997
Using dentures	0.65 (0.36–1.15)	0.139	0.74 (0.47–1.16)	0.185	0.49 (0.26–0.95)	0.035
Choking easily	0.71 (0.16–3.04)	0.641	1.26 (0.46–3.45)	0.653	2.25 (0.65–7.74)	0.199
One item of the Vitality Index						
Low motivation	1.42 (0.79–2.57)	0.246	1.58 (1.01–2.47)	0.044	1.43 (0.76–2.69)	0.267
Five items of the DBD-13						
Lack of interest in daily activities	0.45 (0.21–0.97)	0.042	0.65 (0.38–1.11)	0.113	0.81 (0.40–1.66)	0.563
Awaking at midnight	0.81 (0.33–2.03)	0.658	2.21 (1.19–4.10)	0.012	2.09 (0.89–4.91)	0.089
Making an accusation	1.15 (0.45–2.91)	0.769	1.27 (0.65–2.45)	0.483	1.00 (0.40–2.49)	0.999
Walking around	1.47 (0.59–3.67)	0.412	0.80 (0.38–1.71)	0.565	1.28 (0.47–3.52)	0.630
Repeating the same action	0.66 (0.23–1.84)	0.423	1.09 (0.50–2.40)	0.830	1.04 (0.36–2.99)	0.943

Notes: PFT, physical function trajectory; OR, odds ratio; CI, confidence interval; BMI, body mass index; DBD, Dementia Behavior Disturbance. * We used multivariate logistic regression analysis to compare the maintenance group (G1) with each of the other groups, adjusting for a single item of the Barthel index; ‡ low BMI was defined as <18.5 kg/m^2^.

## Data Availability

Data from this study will not be made available to the public because they contain residents’ personal information; however, they are available from the corresponding author on reasonable request.

## Supplementary Materials

The following supporting information can be downloaded at: https://www.mdpi.com/article/10.3390/geriatrics9050123/s1. Table S1: The Barthel index score used in this study; Table S2: Characteristics of LTC facility residents at admission by whether follow-up completed; Figure S1: Trajectory model with two to six groups; Table S3: Fits statistics for PFT models; Figure S2: Actual physical function trajectory for the four-group model; Figure S3: Actual physical function trajectory for the two-to-six-group model.
