# Correction: Ikuta et al. Physical Function Trajectory among High-Functioning Long-Term Care Facility Residents: Utilizing Japanese National Data. *Geriatrics* 2024, *9*, 123

**DOI:** 10.3390/geriatrics11010003

**Published:** 2025-12-30

**Authors:** Kasumi Ikuta, Maiko Noguchi-Watanabe, Miya Aishima, Tatsuhiko Anzai, Kunihiko Takahashi, Sakiko Fukui

**Affiliations:** 1Department of Home Health and Palliative Care Nursing, Tokyo Medical and Dental University, 1-5-45 Yushima, Bunkyo-Ku, Tokyo 113-8519, Japan; 2Department of Biostatistics, M&D Data Science Center, Tokyo Medical and Dental University, 2-3-10 Kandasurugadai, Chiyoda-Ku, Tokyo 101-0062, Japan

## Text Correction

There was an error in the original publication [1]. The patient enrollment period was not specified correctly.

A correction has been made to the below-mentioned sections where the correct patient enrollment period of 1 July 2021 to 28 February 2023 was added. The corrected paragraphs are inserted below:

Abstract:

“The primary outcome was physical function changes after admission. Data were collected from the Long-Term Care Information System for Evidence (LIFE), which monitored LTC facility residents’ function between 1 July 2021 and 28 February 2023.”


*2.1. Study Design, Setting, and Population*


“In this multicenter retrospective cohort study, we utilized routinely collected LIFE data from high-functioning LTC facility residents (Barthel index (BI) > 60 [21]) across 47 urban LTC facilities in Japan between 1 July 2021 and 28 February 2023.”


*3.1. Participant Selection and Follow-Up*


“Of the LTC facility residents admitted between 1 July 2021 and 28 February 2023 (n = 2805, 47 LTC facilities), we excluded residents with a BI ≤ 60 (n = 1539), those with stay periods shorter than six months (n = 548), and those with missing LIFE data (n = 0); thus, we included 718 residents in the analysis (Figure 1).”

Figure 1:

## References

Reference 15 is changed to:

15. Saito, J.; Kondo, N.; Saito, M.; Takagi, D.; Tani, Y.; Haseda, M.; Tabuchi, T.; Kondo, K. Exploring 2.5-Year Trajectories of Functional Decline in Older Adults by Applying a Growth Mixture Model and Frequency of Outings as a Predictor: A 2010–2013 JAGES Longitudinal Study. *J. Epidemiol.* **2019**, *29*, 65–72.

References 23 is also corrected to the following:

23. Shah, S.; Vanclay, F.; Cooper, B. Improving the Sensitivity of the Barthel Index for Stroke Rehabilitation. *J. Clin. Epidemiol.*
**1989**, *42*, 703–709.

The following reference is removed:

Ohura, T.; Higashi, T.; Ishizaki, T.; Nakayama, T. Assessment of the validity and internal consistency of a performance evaluation tool based on the Japanese version of the modified Barthel Index for elderly people living at home. *J. Phys. Ther. Sci.* **2014**, *26*, 1971–1974. 

The statement referring to the removed reference is backed up by reference 24.

With this correction, the order of some references has been adjusted accordingly. The authors state that the scientific conclusions are unaffected. This correction was approved by the Academic Editor. The original publication has also been updated.

## Figures and Tables

**Figure 1 geriatrics-11-00003-f001:**
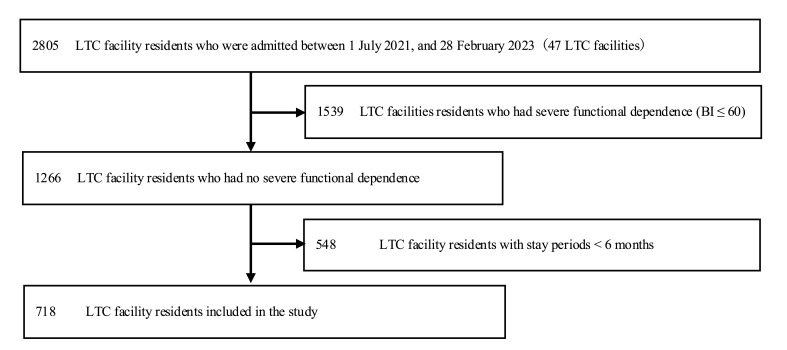
Flow diagram for the inclusion and exclusion of study participants. Notes: LTC, long-term care.
