# Supplementary material for: Physical Function Trajectory among High-Functioning Long-Term Care Facility Residents: Utilizing Japanese National Data
Source: Geriatrics (Basel). 2024 Sep 19;9(5):123. doi: 10.3390/geriatrics9050123 (PMC11417860; doi:10.3390/geriatrics9050123)
Supplement: Supplementary file 1 [file geriatrics-09-00123-s001.zip › Table S1.pdf]

Supplement 1. The Barthel Index score used in this study

Item	Description	Barthel Index score	binarization (self-sufficient: 0, dependence; 1)
Toilet	Dependent	0	1
	Needs some help, but can do some things alone	5	1
	Independent (on and off, dressing, wiping)	10	0
Chair/bed transfer	Unable	0	1
	Major help (1 or 2 people, physical)	5	1
	Minor help (verbal of physical)	10	1
	Independent	15	0
Personal hygiene	Needs help with personal care	0	1
	Independent face/hair/teeth/shaving (implements provided)	5	0
Dressing	Dependent	0	1
	Needs help but can do about half unaided	5	1
	Independent (including buttons, zips, laces, etc.)	10	0
Ambulation	Immobile	0	1
	Wheelchair independent, including corners	5	1
	Walks with help of 1 person (verbal or physical)	10	1
	Independent (but may use any aid)	15	0
Feeding	Unable	0	1
	Needs help cutting, spreading butter, etc. or Requires modified diet	5	1
	Independent	10	0
Bowel control	Incontinent (or needs to be given enemas)	0	1
	Occasional accident (less than once a week)	5	1
	continent	10	0
Bladder control	Incontinent, or catheterized and unable to manage alone	0	1
	Occasional accident (maximal once per 24 h)	5	1
	Continent	10	0
Self-bathing	Dependent	0	1
	Independent (or in shower)	5	0
Stair climbing	Unable	0	1
	Needs help (verbal, physical, carrying aid)	5	1
	Independent	10	0
