# Supplementary material for: Physical Function Trajectory among High-Functioning Long-Term Care Facility Residents: Utilizing Japanese National Data
Source: Geriatrics (Basel). 2024 Sep 19;9(5):123. doi: 10.3390/geriatrics9050123 (PMC11417860; doi:10.3390/geriatrics9050123)
Supplement: Supplementary file 1 [file geriatrics-09-00123-s001.zip › Table S2.pdf]

Supplement 2. Characteristics of LTC facility residents at admission by whether follow-up completed.

	Not complete follow-up	Complete follow-up	P-value
N	548	718	
BMI (mean (SD))	21.58 (21.84)	20.73 (3.18)	0.304
Age (mean (SD))	84.07 (9.12)	85.69 (7.01)	<0.001
Barthel Index at admission (mean (SD))	83.33 (10.75)	82.11 (10.61)	0.044
Single item of Barthel Index			
Feeding (%)	7 (1.3)	19 (2.6)	0.133
Chair/bed transfer (%)	98 (17.9)	125 (17.4)	0.885
Personal hygiene (%)	97 (17.7)	124 (17.3)	0.9
Toilet (%)	413 (75.4)	578 (80.5)	0.033
Self-bathing (%)	420 (76.6)	580 (80.8)	0.085
Ambulation (%)	150 (27.4)	198 (27.6)	0.986
Stair climbing (%)	146 (26.6)	223 (31.1)	0.099
Dressing (%)	90 (16.4)	126 (17.5)	0.651
Bowel control (%)	79 (14.4)	77 (10.7)	0.058
Bladder control (%)	183 (33.4)	298 (41.5)	0.004
History of aspiration pneumonia (%)	7 (1.3)	7 (1.0)	0.811
Low cognitive function (%)	345 (63.0)	492 (68.5)	0.044
Eating preference (soft food) (%)	63 (11.5)	76 (10.6)	0.672
Using dentures (%)	212 (38.7)	289 (40.3)	0.613
Choke easily (%)	26 (4.7)	36 (5.0)	0.929
One item of Vitality index			
Low motivation (%)	261 (47.6)	300 (41.8)	0.044
Five items of the DBD-13			
Lack of interest in daily activities (%)	147 (26.8)	196 (27.3)	0.901
Awaking at midnight (%)	99 (18.1)	121 (16.9)	0.624
Making an accusation (%)	73 (13.3)	98 (13.6)	0.931
Walking around (%)	140 (25.5)	159 (22.1)	0.178
Repeating the same action (%)	103 (18.8)	122 (17.0)	0.449

LTC, long-term care; SD, standard deviation; BMI, body mass index; DBD, Dementia Behavior Disturbance
