# Supplementary material for: Physical Function Trajectory among High-Functioning Long-Term Care Facility Residents: Utilizing Japanese National Data
Source: Geriatrics (Basel). 2024 Sep 19;9(5):123. doi: 10.3390/geriatrics9050123 (PMC11417860; doi:10.3390/geriatrics9050123)
Supplement: Supplementary file 1 [file geriatrics-09-00123-s001.zip › Table S3.pdf]

Supplement 4. Fits statistics for PFT models

Group	Degree	N	Group proportion	AvePP	OCC*	AIC	BIC	logLik
2	3	474	0.398	1	-	-658113.5	-658075.4	330087.7
		244	0.205	1	-			
3	3	474	0.398	1	-	-659493.0	-659423.2	330737.3
		185	0.155	0.989	484.9			
		59	0.049	0.966	552.3			
4	3	474	0.398	1.000	-	-660129.2	-660027.6	331039.4
		68	0.057	0.984	997.5			
		119	0.100	0.977	378.0			
		57	0.048	0.988	1612.2			
5	3	474	0.398	1.000	-	-660544.3	-660417.4	331232.4
		62	0.052	0.950	349.2			
		67	0.056	0.979	790.5			
		46	0.039	0.982	1329.1			
		69	0.058	0.993	2342.5			
6	3	474	0.398	1.000	-	-660576.4	-660411.4	331245.2
		56	0.047	0.993	2797.2			
		69	0.058	0.993	2412.9			
		69	0.058	0.940	254.5			
		8	0.007	0.926	1862.9			
		42	0.035	0.935	391.9			

PFT, physical function trajectory; AvePP, average posterior probability; OCC, odds of correct classification; AIC, Akaike's information criterion; BIC, Bayesian information criterion

* OCC: The numerator is the odds of a correct classification into a certain group based on the model, and the denominator is the correct classification into that group based on random assignment, essentially, OCC = [b/(1-b)]/[a/(1-a)].

For each trajectory group, OCC >= 5 suggests high assignment accuracy
