# Supplementary figures and images for: Physical Function Trajectory among High-Functioning Long-Term Care Facility Residents: Utilizing Japanese National Data
Source: Geriatrics (Basel). 2024 Sep 19;9(5):123. doi: 10.3390/geriatrics9050123 (PMC11417860; doi:10.3390/geriatrics9050123)

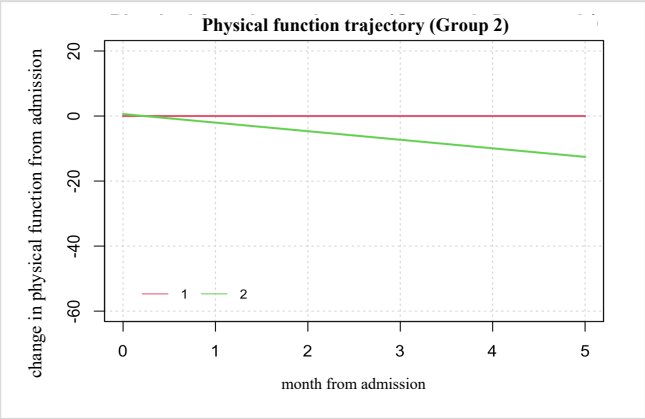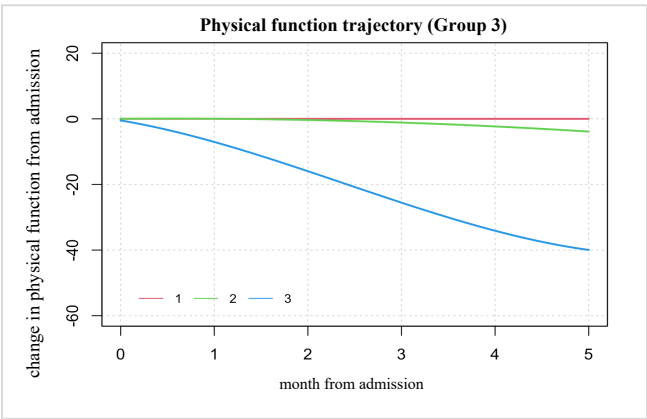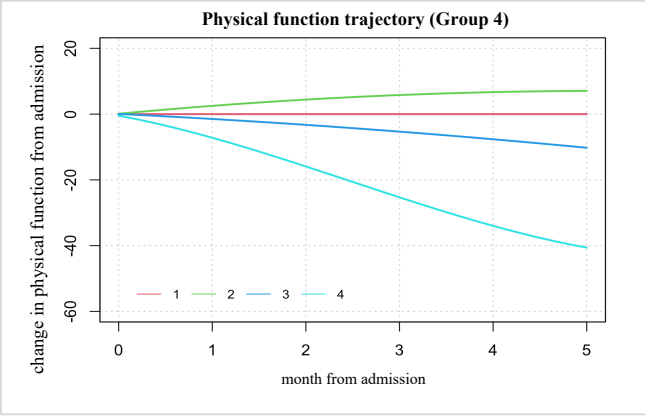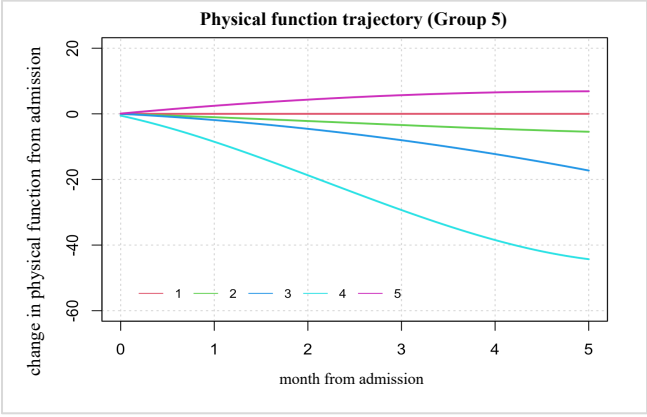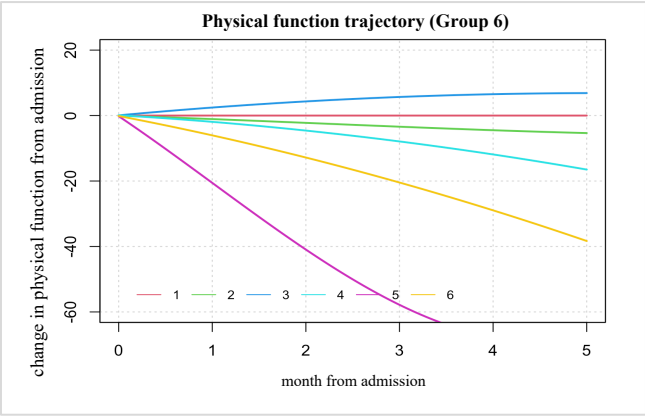

Supplement: Supplementary file 1 [file geriatrics-09-00123-s001.zip › Figure S1.pdf]

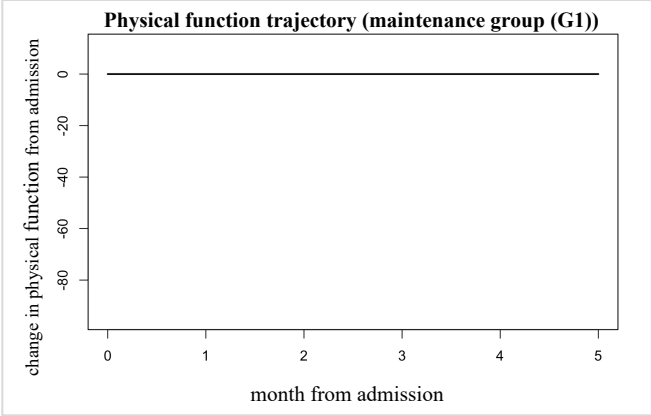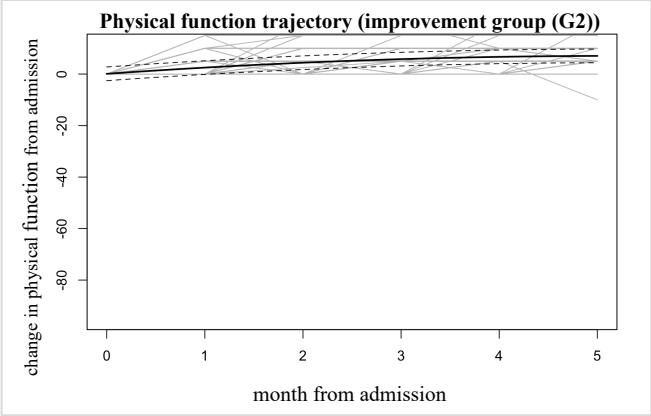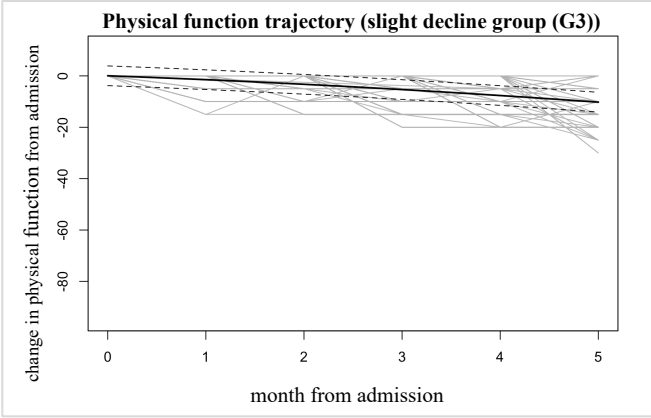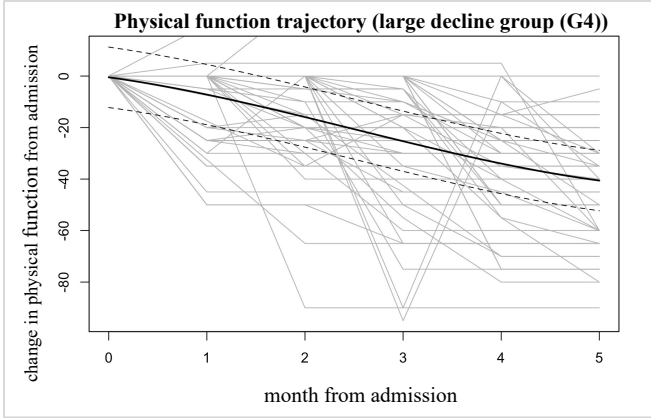

Supplement: Supplementary file 1 [file geriatrics-09-00123-s001.zip › Figure S2.pdf]

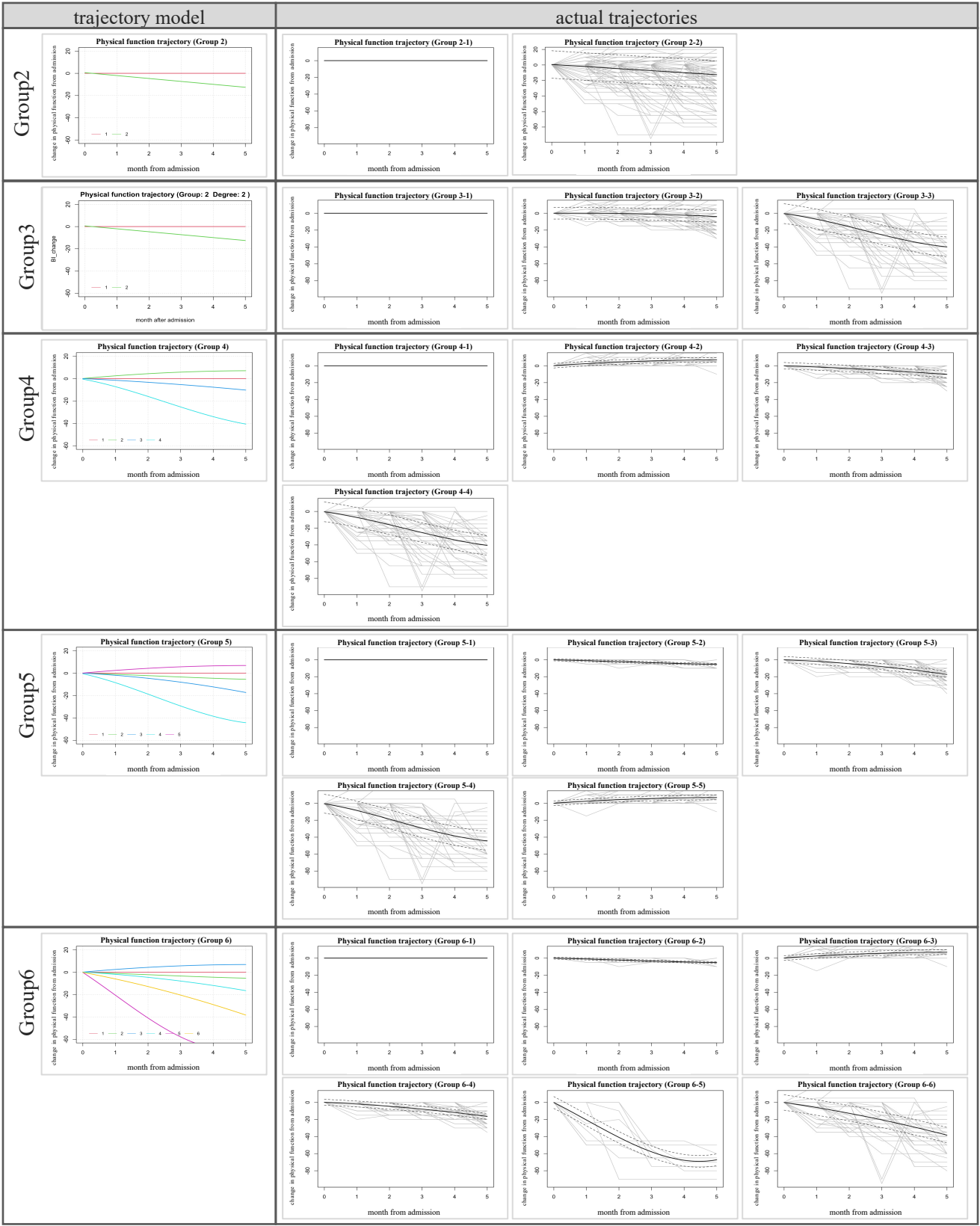

Supplement: Supplementary file 1 [file geriatrics-09-00123-s001.zip › Figure S3.pdf]
